# Fermi surface and kink structures in $\hbox {Sr}_{{4}}\hbox {Ru}_{{3}}\hbox {O}_{{10}}$ revealed by synchrotron-based ARPES

**DOI:** 10.1038/s41598-020-77845-x

**Published:** 2020-12-03

**Authors:** Prosper Ngabonziza, Emanuela Carleschi, Volodymyr Zabolotnyy, Amina Taleb-Ibrahimi, François Bertran, Rosalba Fittipaldi, Veronica Granata, Mario Cuoco, Antonio Vecchione, Bryan Patrick Doyle

**Affiliations:** 1 Max Planck Institute for Solid State Research, Heisenbergstraße 1, 70569 Stuttgart, Germany; 2 Department of Physics, University of Johannesburg, PO Box 524, Auckland Park , 2006 South Africa; 3 Physikalisches Institut, Julius-Maximilians-Universität Würzburg, Am Hubland, 97074 Würzburg, Germany; 4 Synchrotron SOLEIL, L’Orme des Merisiers, Saint-Aubin-BP48, 91192 Gif-sur-Yvette, France; 5 CNR-SPIN Salerno, Via Giovanni Paolo II, 84084 Fisciano, Italy; 6 Department of Physics, University of Salerno, Via Giovanni Paolo II, 84084 Fisciano, Italy

**Keywords:** Physics, Condensed-matter physics

## Abstract

The low-energy electronic structure, including the Fermi surface topology, of the itinerant metamagnet $\hbox {Sr}_{{4}}\hbox {Ru}_{{3}}\hbox {O}_{{10}}$ is investigated for the first time by synchrotron-based angle-resolved photoemission. Well-defined quasiparticle band dispersions with matrix element dependencies on photon energy or photon polarization are presented. Four bands crossing the Fermi-level, giving rise to four Fermi surface sheets are resolved; and their complete topography, effective mass as well as their electron and hole character are determined. These data reveal the presence of kink structures in the near-Fermi-level band dispersion, with energies ranging from 30 to 69 meV. Together with previously reported Raman spectroscopy and lattice dynamic calculation studies, the data suggest that these kinks originate from strong electron–phonon coupling present in $\hbox {Sr}_{{4}}\hbox {Ru}_{{3}}\hbox {O}_{{10}}$. Considering that the kink structures of $\hbox {Sr}_{{4}}\hbox {Ru}_{{3}}\hbox {O}_{{10}}$ are similar to those of the other three members of the Ruddlesden Popper structured ruthenates, the possible universality of strong coupling of electrons to oxygen-related phonons in $\hbox {Sr}_{n+1}\hbox {Ru}_{{n}}\hbox {O}_{3n+1}$ compounds is proposed.

## Introduction

Strontium ruthenate oxides of the Ruddlesden Popper (RP) series, $\hbox {Sr}_{n+1}\hbox {Ru}_{{n}}\hbox {O}_{3n+1}\, (n= 1, 2, 3, \infty )$, are a class of materials in the realm of 4*d* transition metal oxides that are attractive for both fundamental and applied research. From the perspective of fundamental physics, $\hbox {Sr}_{n+1}\hbox {Ru}_{{n}}\hbox {O}_{3n+1}$ are strongly correlated materials which exhibit complex interplay between the charge, spin, orbital and lattice degrees of freedom^1^. Their physical properties depend on the change of the number *n* of the $\hbox {RuO}_{{6}}$ octahedra layers in the unit cell; and they range from the unconventional superconductivity in $\hbox {Sr}_{{2}}\hbox {RuO}_{{4}}$ ($n=1$)^2,3^, the quantum critical metamagnetism and nematicity in $\hbox {Sr}_{{3}}\hbox {Ru}_{{2}}\hbox {O}_{{7}}$ $(n=2)$^4–6^, anisotropic ferromagnetism and proposed orbital-dependent metamagnetism in $\hbox {Sr}_{{4}}\hbox {Ru}_{{3}}\hbox {O}_{{10}}$ (n=3)^7–10^ and the spontaneous itinerant ferromagnetism in $\hbox {SrRuO}_3\,(n=\infty )$^11^. On the device applications side, members of RP-structured strontium ruthenates have been integrated in oxide electronic devices due to their good stability and structural compatibility with other correlated oxides. For example, $\hbox {SrRuO}_3$ has been widely used as a conductive electrode in diverse oxide devices and heterostructures/superlattices^12^. $\hbox {SrRuO}_3/\hbox {Sr}_{{2}}\hbox {RuO}_{{4}}$ hybrid structures have been explored in tunnel junction devices to establish superconducting spintronics utilizing ferromagnet/superconductor heterostructures^13^. Also, the $\hbox {Sr}_{{3}}\hbox {Ru}_{{2}}\hbox {O}_{{7}}/\hbox {SrRuO}_3$ and $\hbox {Sr}_{{4}}\hbox {Ru}_{{3}}\hbox {O}_{{10}}/\hbox {SrRuO}_3$ heterostructures have been reported to grow epitaxially on conventional oxide substrates^14^, which could pave a way for the precise exploration of the known size effects in the magnetic properties of these compounds^15^ by using ultra-thin devices fabricated from such heterostructures. All these behaviours show that $\hbox {Sr}_{n+1}\hbox {Ru}_{{n}}\hbox {O}_{3n+1}$ materials provide a fascinating playground to explore both novel quantum phenomena and diverse oxide-based electronic device directions.

We focus on the three layered member of the strontium ruthenate series. The triple-layered, $\hbox {Sr}_{{4}}\hbox {Ru}_{{3}}\hbox {O}_{{10}}$, is a quasi-two-dimensional metal with an orthorhombic unit cell, which has attracted attention due to its unique magnetic properties that are marked by the coexistence of ferromagnetism and itinerant metamagnetism^7–9,16^. For a field applied along the *c* axis $(H\parallel c)$, $\hbox {Sr}_{{4}}\hbox {Ru}_{{3}}\hbox {O}_{{10}}$ displays a ferromagnetic transition with a Curie temperature of  $T_{\text {Curie}}\approx 105$ K, followed by another sharp transition at $T_{\text {M}}\approx 60$ K that is accompanied by a resistive anomaly in transport measurements^7–9,17,18^. For temperatures below $T_{\text {M}}$, the application of a magnetic field within the *ab* plane $(H\parallel ab)$ induces a metamagnetic transition (i.e., a superlinear increase in the magnetization) at about 2.5 T^10,16^. In addition, double metamagnetic behaviour was recently reported in magnetization data of $\hbox {Sr}_{{4}}\hbox {Ru}_{{3}}\hbox {O}_{{10}}$ with the second metamagnetic transition at a critical field slightly larger than the main metamagnetic jump^19^. However, the physical mechanisms of the exotic magnetic properties of $\hbox {Sr}_{{4}}\hbox {Ru}_{{3}}\hbox {O}_{{10}}$ below $T_{\text {M}}$ still are not well understood and remain elusive. For example, early specific-heat data exhibited no anomaly around $T_{\text {M}}$, which indicates that this second transition may not be an actual thermodynamic phase transition^20^; whereas recent magnetoresistance measurements suggested that the second magnetic transition at $T_{\text {M}}$ originates from a spin reorientation^21^. The metamagnetic transition was suggested to be an orbital-dependent effect, originating from the Ru${\,4d}$ magnetic moments, where the Ru${\,4d_{xz,yz}}$ orbital is responsible for the metamagnetic transition while the $4d_{xy}$ orbital is ferromagnetic in the ground state^7,16^. On the other hand, to explain the double metamagnetic transition, it has been proposed that the metamagnetic behaviours could also originate either from magnetic ordering of the two inequivalent Ru sites or from fine structure in the near Fermi level ($E_F$) electronic structure due to the presence of two van Hove singularities (vHS) in the density of states (DOS)^19^. These vHS would render the material magnetically unstable to the extent that a metamagnetic transition would be induced by the application of a magnetic field.

Although the magnetic properties of $\hbox {Sr}_{{4}}\hbox {Ru}_{{3}}\hbox {O}_{{10}}$ have been extensively investigated^7–10,15–31^ and a possible origin of magnetic fluctuations suggested to be the presence of flat bands near-$E_F$ in the electronic band dispersions, i.e., the vHS^19^, as already predicted^32,33^ and experimentally confirmed^34,35^ for its parent compound $\hbox {Sr}_{{3}}\hbox {Ru}_{{2}}\hbox {O}_{{7}}$; little is know about the electronic band structure of $\hbox {Sr}_{{4}}\hbox {Ru}_{{3}}\hbox {O}_{{10}}$. Thus, a precise characterization of the electronic band structure of this material by means of experimental probes is highly desirable. Angle-resolved photoemission spectroscopy (ARPES) is the best candidate because it is one of the most direct methods to measure the momentum-dependent electronic band dispersion of solids^36^.

Here, we report the first synchrotron-based ARPES measurements on the near-$E_F$ band structure of $\hbox {Sr}_{{4}}\hbox {Ru}_{{3}}\hbox {O}_{{10}}$, including its Fermi surface (FS) topology. Firstly, we present experimental FS maps. From a careful and systematic analysis of the individual quasiparticle bands that make up the FS maps, we are able to identify four FS sheets in the first Brillouin zone (BZ) of $\hbox {Sr}_{{4}}\hbox {Ru}_{{3}}\hbox {O}_{{10}}$. Secondly, taking advantage of the wide spectral range with an intense and highly polarized continuous spectrum offered by synchrotron radiation facilities, we study the effect of changing different matrix elements on the electronic band dispersions and FS maps of $\hbox {Sr}_{{4}}\hbox {Ru}_{{3}}\hbox {O}_{{10}}$. Thirdly, we study the coupling of the electronic degrees of freedom with collective excitations arising from magnetic or phononic modes. We resolve five kinks in the near-$E_F$ band dispersions with energies ranging from 30 to 69 meV. A detailed analysis of kink energies and their comparison with reported Raman and lattice dynamic calculations data suggest that the kinks originate from strong electron-phonon coupling present in this system. By referring to other members of the strontium ruthenate oxides of the RP series exhibiting similar kink structure, we infer that the electron-phonon correlations may be ubiquitous in $\hbox {Sr}_{n+1}\hbox {Ru}_{{n}}\hbox {O}_{3n+1}$ compounds. We finish with a discussion on flat bands close to the Fermi-level that could be the electronic origin of magnetic instabilities in this system and suggest that electronic structure calculations, as well as further ARPES measurements, are needed to explore further the link between the fine structure in the near-$E_F$ electronic structure and magnetic instabilities present in the $\hbox {Sr}_{{4}}\hbox {Ru}_{{3}}\hbox {O}_{{10}}$ material.

## Methods and experimental details

Single crystals of $\hbox {Sr}_{{4}}\hbox {Ru}_{{3}}\hbox {O}_{{10}}$ were grown using the flux-feeding floating zone technique with Ru self-flux. Details on the growth procedure of the samples used in this study are reported in Refs.^37,38^. The structural quality, composition and magnetic as well as electrical properties of the single crystals were characterised by X-ray diffraction, energy dispersive microscopy, scanning electron microscopy, specific heat, resistivity and susceptibility measurements. Samples were found to be pure single crystals of $\hbox {Sr}_{{4}}\hbox {Ru}_{{3}}\hbox {O}_{{10}}$.

All ARPES data on $\hbox {Sr}_{{4}}\hbox {Ru}_{{3}}\hbox {O}_{{10}}$ presented here were taken at the beamline CASSIOPÉE of the synchrotron facility SOLEIL using a Scienta R4000 electron energy analyzer, which allowed us to measure simultaneously many energy distribution curves (EDC) in an angular range of 30$^\circ$. Two single crystals of $\hbox {Sr}_{{4}}\hbox {Ru}_{{3}}\hbox {O}_{{10}}$, labeled as sample $A_1$ and sample $A_2$, of dimensions of approximately $1\times 1 \times 0.5$ $\hbox {mm}^3$ were used during the experiment. Experimental results were found to be reproducible for these two samples. The samples were cleaved in situ at a pressure of $\sim 2 \times 10^{-10}$ mbar and kept at a constant temperature of 5 K throughout the experiment. The spectra presented here are constituted of high resolution *k*-space band dispersions and FS maps of $\hbox {Sr}_{{4}}\hbox {Ru}_{{3}}\hbox {O}_{{10}}$ taken at photon energies of 37 eV, 60 eV and 110 eV. The angular resolution was 0.1$^\circ$ and the nominal energy resolution was 15 meV. The polarization of the incoming light was exploited in order to perform polarization-dependent ARPES experiments. Samples were mounted on the sample holder in such a way that their square in-plane crystallographic axes were aligned with the horizontal and vertical polarization of the incoming photon beam. In this way, FS maps and high-resolution cuts are symmetric, and unrotated in the first BZ, thus facilitating the identification of different matrix elements on the measured FS maps and high-resolution cuts.

## Results and discussions

## Fermi surface of $\hbox {Sr}_{{4}}\hbox {Ru}_{{3}}\hbox {O}_{{10}}$

**Figure 1:**
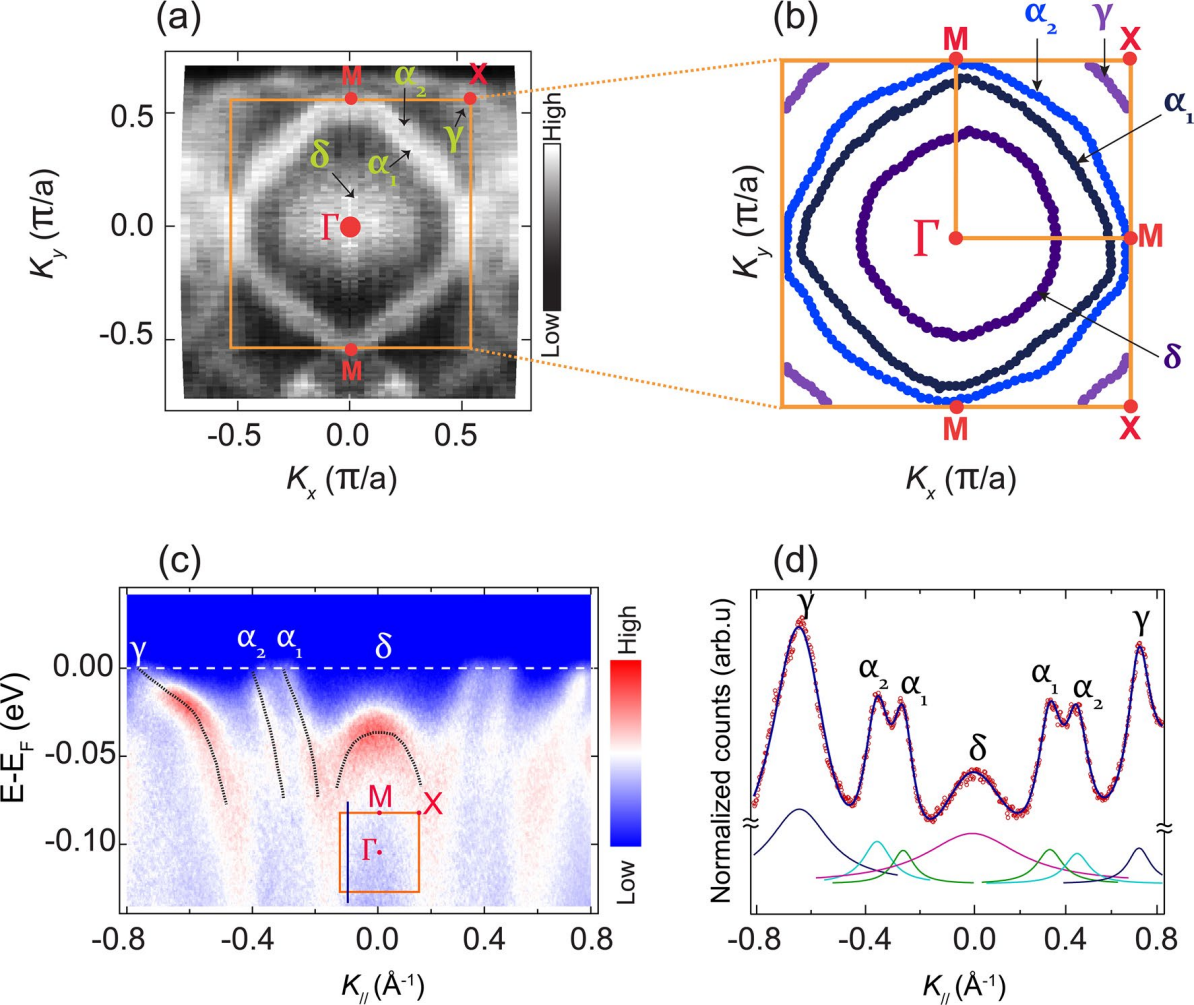
(**a**) FS map of $\hbox {Sr}_4\hbox {Ru}_3\hbox {O}_{{10}}$ for the sample $A_1$ measured using a photon energy of 60 eV in linear vertical polarization. The bright features are regions where the bands cross $E_F$. The experimentally determined first BZ (orange square) and the positions of the high symmetry points are indicated. The FS map has been symmetrized with respect to the vertical line joining two *M* points passing through the $\Gamma$ point. (**b**) Unsymmetrised contour map of the FS of $\hbox {Sr}_4\hbox {Ru}_3\hbox {O}_{{10}}$ extracted from the original FS map data. Four FS sheets $(\delta , \alpha _1, \alpha _2, \text { and }\gamma )$ are identified in the contour map extracted from the analysis of individual cuts making up the FS map. The ${{K}}_{_{//}}$ values in (**a**) and (**b**) are given in units of $\pi /a$, where *a* is the in-plane crystalline constant of $\hbox {Sr}_4\hbox {Ru}_3\hbox {O}_{{10}}$. (**c**) A representative ARPES cut. The blue vertical line in the inset orange BZ indicates the direction in which the cut was acquired. The black points overlaying the dispersing bands are extracted band dispersions for the $\hbox {E}_{\text {F}}$ crossing bands that give rise to the resolved FS sheets, which were clearly identified by fitting several MDCs. The slight intensity asymmetry visible around $\text {K}_{_{//}}=0$ could be ascribed to a misalignment of $\pm 1^{\circ }$ during the mounting of the sample on the sample holder. (**d**) A representative MDC spectrum in red symbols together with the overall fitting (solid blue line) performed with Lorentzian line shapes. The individual Lorentzian peaks for the bands of interest are also shown.

Figure 1a depicts a FS map covering slightly more than the first BZ of $\hbox {Sr}_{{4}}\hbox {Ru}_{{3}}\hbox {O}_{{10}}$. A nominal energy resolution of 15 meV was used and data have been integrated over an energy window of $\pm 12$ meV around $E_F$. The bright features correspond to regions where the bands cross $E_F$. Since it is the first time FS maps of $\hbox {Sr}_{{4}}\hbox {Ru}_{{3}}\hbox {O}_{{10}}$ are reported, the resolved FS sheets were labelled keeping notations consistent with the previously resolved FS sheets of $\hbox {Sr}_{{2}}\hbox {RuO}_{{4}}$^39^ and $\hbox {Sr}_{{3}}\hbox {Ru}_{{2}}\hbox {O}_{{7}}$^34^. For the assignment of the *M* and *X* points in the surface first BZ of $\hbox {Sr}_{{4}}\hbox {Ru}_{{3}}\hbox {O}_{{10}}$, we followed the notation consistent with the reported square BZ of $\hbox {Sr}_{{2}}\hbox {RuO}_{{4}}$^39–41^. This is because our low temperature (5 K) ARPES data on the FS maps of $\hbox {Sr}_{{4}}\hbox {Ru}_{{3}}\hbox {O}_{{10}}$ suggest that its first BZ is not rotated as in the single layer system, $\hbox {Sr}_{{2}}\hbox {RuO}_{{4}}$; unlike the double layered system $\hbox {Sr}_{{3}}\hbox {Ru}_{{2}}\hbox {O}_{{7}}$, which has a rotated square BZ^34^.

We have resolved four FS sheets from the experimental ARPES measured FS map (Fig. 1a). The round contour centred at the $\Gamma$ point is identified as the $\delta$ FS sheet. A closer look at the map reveals three more FS sheets: two larger FS sheets centred around the $\Gamma$ point that are labelled $\alpha _1$ and $\alpha _2$, respectively; and a smaller one centred around the *X* points, labelled $\gamma$. To illustrate the approximate shape of the resolved FS sheets, a contour map extracted from this map by fitting peaks from individual high resolution cuts making up the FS map is presented in Fig. 1b. The $E_F$ crossings of the four bands that give rise to these FS sheets have been clearly identified through the analysis of high resolution cuts taken at different *k*-directions in the BZ. Fig. 1c gives a representative ARPES cut taken in the direction parallel to the $\Gamma -M$ line in the first BZ. To follow the dispersion of these bands from $E_F$, several equally spaced momentum dispersive curves (MDCs) have been fitted with Lorentzian functions and the extracted peak positions are plotted on top of the corresponding band (black dashed lines) in the ARPES cut. Figure 1d shows a representative MDC taken at $E_F$ and fitted with Lorentzian functions. From the MDC analyses, the slopes of the $\alpha _1, \alpha _2$ and $\gamma$ bands at $E_F$ are calculated to be 0.58 eV.Å, 1.14 eV.Å  and 0.16 eV.Å, respectively. From these slopes, the effective masses have been calculated for individual FS sheets using Fermi velocities determined along different band dispersions. A detailed analysis of these four $E_F$-crossing bands indicate that the $\gamma \text { and } \delta$ bands have hole-like character, whereas the $\alpha$ bands have electron-like character (Supplementary Table S1, Supplementary Figure  S2).

Considering a tetragonal crystal structure, a single trilayer of $\hbox {RuO}_6$ octahedra contains 3 $\hbox {Ru}^{4+}$ ions each contributing 4 conducting electrons distributed over the 3 nearly degenerate $t_{2g}$ levels^8,19^. In a first approximation; from the electron count, considering that there are small orthorhombic distortions and magnetic exchanges are small, it is expected to have 3 Ru $4d\,\,t_{2g}\times 3$ layers in the unit cell resulting in 9 electrons that would then give rise to nine FS sheets. However, from our ARPES data, we have resolved only 4 bands crossing the $E_F$ giving rise to four FS sheets. One possible reason for these undetected bands could be the inter-layer splitting mechanism in this system. As indicated above, in the consideration of small orthorhombic distortions and small magnetic exchanges, we are left with bonding, non-bonding and anti-bonding type of bands due to the layer degree of freedom. Since the $d_{xy}$ bands are not dispersive along the *z*-axis, they will be degenerate such that 3 out of the 9 bands will make one FS sheet with *xy* character. The remaining bands originating from the (*xz*, *yz*) orbitals will form bonding, anti-bonding and non-bonding bands due to the out-of-plane hybridization with a splitting of the order of 150 meV^1,42^. Since the (*xz*, *yz*) orthorhombic splitting is small (below the resolution limit), we would then end up with 3 bands (2-fold degenerate) with (*xz*, *yz*) character and 1 band (3-fold degenerate) with *xy* character; thus a total of 4 bands at $E_F$ giving rise to four FS sheets. An alternative reason for undetected bands could be the experimental energy resolution, nominally 15 meV. It could be that there are more bands very close to each other that contribute to the same ARPES peak which are not experimentally resolved. This argument is supported by previous ARPES data on $\hbox {Sr}_3\hbox {Ru}_2\hbox {O}_{{7}}$ in which an effective experimental resolution of $\sim 5.5$ meV was used to resolve all the expected $E_F$-crossing bands^34^, suggesting that a complete determination of all $E_F$ crossing bands in $\hbox {Sr}_4\hbox {Ru}_3\hbox {O}_{{10}}$ is resolution limited. For more quantitative analysis and confirmation of these hypotheses with respect to the observed FS sheets, electronic structure calculations are indispensable, taking into account all the degrees of freedom present in this complex 4*d* system.

## Matrix element effects in photoemission spectra of $\hbox {Sr}_{{4}}\hbox {Ru}_{{3}}\hbox {O}_{{10}}$

To probe different features of the near-$E_F$ band dispersions, we have explored the effect of matrix elements on the quasiparticle band dispersions of $\hbox {Sr}_4\hbox {Ru}_3\hbox {O}_{{10}}$ using different photon energies (60 and 110 eV) and polarizations of the incident light, linear horizontal polarization (LHP) and linear vertical polarization (LVP). A detailed discussion on photoemission matrix elements is presented in the Supporting Information (Fig. S3a–c) and Refs.^43–45^. Exploiting the energy and polarization dependence of the ARPES matrix elements have already been demonstrated to be a powerful tool to extract the symmetry properties of the electronic states and disentangle the primary contributions of the main bands to the spectrum from those arising from secondary effects^44,46,47^.

**Figure 2:**
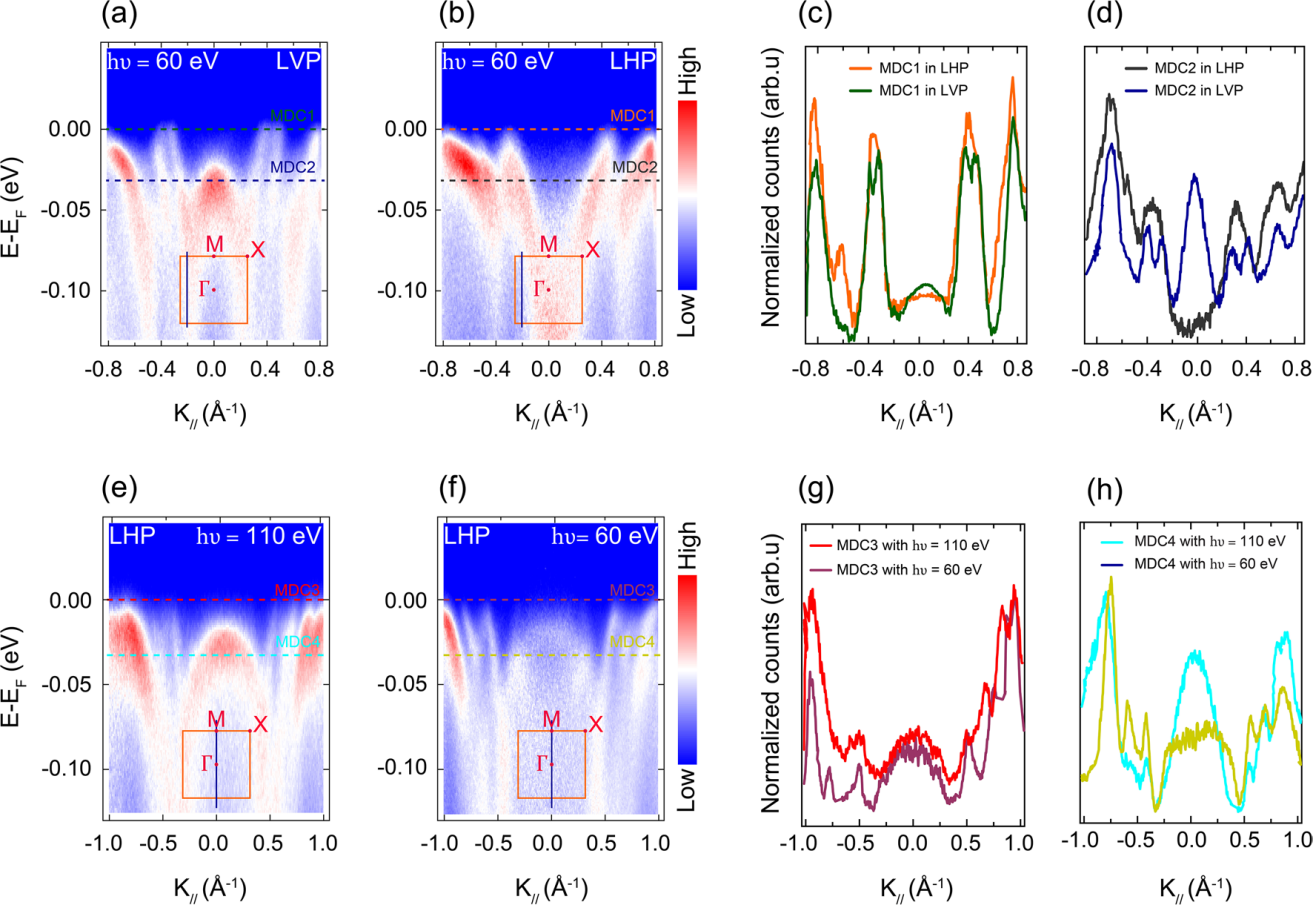
Photoemission matrix element effects on near-$E_F$ band dispersions of $\hbox {Sr}_4\hbox {Ru}_3\hbox {O}_{{10}}$ acquired at different locations in the BZ. Different features were resolved in ARPES spectra taken at the same location in the BZ with the same photon energy of 60 eV, but using different light polarizations (**a**) LVP (**b**) LHP; and with the same light polarization (LHP), but using different photon energies (**e**) 110 eV and (**f**) 60 eV. The blue vertical lines in the orange square BZ (insets) show the direction in which the cuts were acquired. The horizontal dashed lines indicate the energy positions (at $E_F$ and 30 meV) on which the MDCs in (**c**,**d**) and (**g**,**h**) were extracted. All spectra are from the sample $A_1$.

To exploit the effects of using different polarizations of the incident light, several ARPES cuts were taken using the same photon energy in the same position of the BZ, but with different light polarizations. Figure 2a,b show representative ARPES band dispersions taken with the same photon energy of 60 eV in LVP and LHP, respectively. The band centred around the $\Gamma$ point is clearly resolved for the ARPES cut in LVP, while for the one in LHP the same band is not resolved at all; which is further confirmed by MDCs extracted at different locations on these ARPES cuts (Fig. 2c,d). Moreover, for the ARPES cut taken in LVP, there are two clearly different bands, labelled $\alpha _1$ and $\alpha _2$ that are well resolved (Fig. 2a), whereas in LHP these two bands form one band. One can not differentiate their dispersions in the ARPES cut in LHP (Fig. 2b), as clearly shown by the extracted MDCs (Fig. 2c,d). Fitting MDCs extracted from this ARPES cut at $E_F$, with Lorentzian functions (Fig. 1d), the exact separation between the $\alpha _1$ and $\alpha _2$ bands is determined to be $\Delta K_{\parallel E_f}\simeq 0.102\pm 0.001$ Å. We have observed the same splitting for the FS maps acquired with incoming synchrotron light in LVP. Two FS sheets $\alpha _1$ and $\alpha _2$ that are centred around the $\Gamma$ point were resolved in LVP while only one FS sheet was resolved in LHP (see Supplementary Figure S3d,e).

From the ARPES spectra acquired at different light polarizations, we have analysed the in-plane and out-of-plane band character of the $\hbox {Sr}_4\hbox {Ru}_3\hbox {O}_{{10}}$ system. When light is in LHP, it is possible to probe both in-plane bands and out-of-plane bands by rotating the sample by a certain polar angle $\varphi$ around the vertical axis; while in LVP, one would only probe in-plane bands (see details in Supporting Information and Supplementary Figure  S3c). From the two ARPES spectra shown in Fig. 2a,b, we speculate that the $\alpha _1$ and $\alpha _2$ bands have in-plane character ($d_{xy}, d_{x^2-y^2}$) since they are better resolved to be two separate bands when light is in LVP; while in LHP, they merge in one broader band. For the other resolved bands, the exploitation of light polarization together with experimental geometry showed no effect on their in-plane and out-of-plane characters; thus, band structure calculations of $\hbox {Sr}_4\hbox {Ru}_3\hbox {O}_{{10}}$ are therefore desirable to determine the character of all experimentally observed bands.

Furthermore, several ARPES cuts were taken using different photon energies, and their photoemission intensity was investigated. Figure 2e,f show two ARPES cuts which were both acquired in LHP, but using different photon energies of 60 and 110 eV, respectively. These two energies were chosen as they showed the largest effects. Comparing these two ARPES band dispersions, we have observed that in general photoemission intensity is more enhanced for the ARPES cut taken with a photon energy of 110 eV. In particular, focusing on the band that gives rise to the $\delta$ FS sheet around the $\Gamma$ point, it is observed that for $h\nu = 110$ eV the photoemission intensity is more enhanced and the band is better resolved. However, for $h\nu = 60$ eV, the intensity of this same band is diminished and the band is not clearly resolved, as also demonstrated in the extracted MDCs in Fig. 2g,h. This observation shows the advantage of matrix elements to enhance the photoemission intensity of some band features while suppressing the intensity of others in electronic band dispersion.

## Electron–phonon interactions in band structure of $\hbox {Sr}_{{4}}\hbox {Ru}_{{3}}\hbox {O}_{{10}}$

The interaction of electrons with other excitations, e.g., phonons or spin excitations, often leads to anomalies in the energy–momentum dispersion relations that result in electronic band renormalization and sudden changes, referred to as *kinks*, in the slope of the near-$E_F$ band dispersions^48–52^. The kink energies are related to the relevant energy scales present in the investigated system, such as the coupling of electrons with other excitations. Kink structures have been reported in ARPES spectra at energies within 100 meV of $E_F$ in many correlated oxide systems, such as various cuprate superconductors^51,53^, $\hbox {SrVO}_3$^52^ and the RP-structured manganite $\hbox {La}_{2-2x}\hbox {Sr}_{1+2x}\hbox {Mn}_2\hbox {O}_7$^54^. Here, we study kink structures in the electronic band dispersion of $\hbox {Sr}_4\hbox {Ru}_3\hbox {O}_{{10}}$.

**Figure 3:**
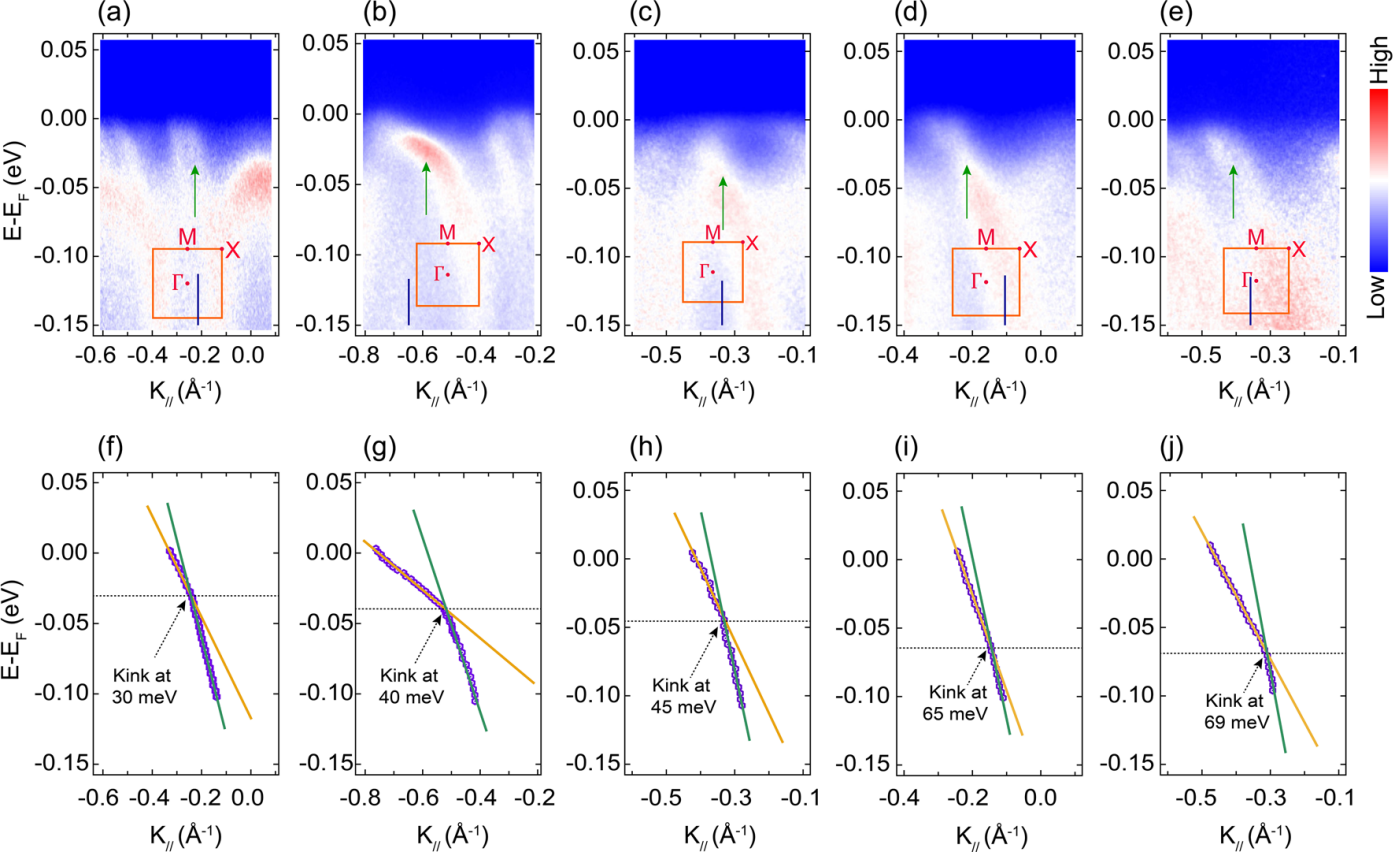
Kinks in the band dispersions of $\hbox {Sr}_4\hbox {Ru}_3\hbox {O}_{{10}}$ for the sample $A_1$. (**a**–**e**) The photoemission intensity spectra measured at different positions in the first Brillouin zone as shown in the inset of each cut. The blue vertical line in the BZ shows the direction in which the cuts were acquired. The green arrow in each cut indicates the band of interest. (**f**–**j**) Band dispersion (purple symbols) extracted from the fitting of MDCs extrapolated from the corresponding cuts in the upper panels for the band of interest. Black dashed arrows point to where kinks are observed, at the intersections of two linear fits to the low energy data. A possible second kink is visible in (**h**) at approximately 19 meV. A plausible origin for this kink is discussed in the final section of the Supporting Information.

Figure 3a–e depict ARPES spectra of $\hbox {Sr}_4\hbox {Ru}_3\hbox {O}_{{10}}$ in selected locations of the BZ that were acquired using a photon energy of 60 eV in LVP. The bands of interest are indicated by green arrows in the top panels of the figure. These bands change slope as they approach $E_F$ and their photoemission intensity distribution varies. Some of the bands are more enhanced and better resolved (Fig. 3b) as they approach $E_F$ than others (Fig. 3a). To quantitatively investigate the behaviour of these bands in the vicinity of $E_F$, several MDCs extracted from these spectra were fitted with Lorentzian functions and the binding energy of the MDC maxima, for the particular band of interest, have been followed down to binding energies of around $-0.1$ eV. Figure 3f-j show the extracted MDC peak maxima as a function of momentum. Kink features are clearly observed in the dispersion of these bands. To extract the kink energies, the near-$E_F$ low energy parts of the experimental dispersions have been fitted with two straight lines passing through the Fermi momentum $k_F$ (green and orange straight lines in Fig. 3f-j). Kink energies of 30, 40, 45, 65 and 69 meV were extracted. This energy range is comparable with what was previously reported in near-$E_F$ dispersions of other Sr-based layered ruthenates^55–59^ and low energy kinks in the high-Tc superconductors^50,60^. The presence of kinks in the electronic band dispersions of $\hbox {Sr}_4\hbox {Ru}_3\hbox {O}_{{10}}$ is further confirmed by the analysis of the MDC full width half maximum (FWHM) versus the binding energy of the peaks (Supplementary Figure  S4). The FWHM of the MDC is directly proportional to the imaginary part of the self energy^61^, which represents the scattering rate of the quasiparticles in the system. The scattering rate for this particular cut shows a discontinuity at an energy of $\sim 43$ meV, which is in very good agreement with the 40 meV kink observed for this particular feature (Supplementary Figure S4c).

To understand the origin of kinks in the band structure of $\hbox {Sr}_4\hbox {Ru}_3\hbox {O}_{{10}}$, the extracted kink energies were compared to previous phonon modes from Raman spectroscopy and lattice dynamic calculations (LDCs)^62,63^. Remarkably, the kink energies have a good correspondence with the energy of the phononic modes from Raman spectroscopy and LDCs (Supplementary Table S2). Thus, it is likely that the coupling of electrons to phonons plays a dominant role in the formation of kinks in $\hbox {Sr}_4\hbox {Ru}_3\hbox {O}_{{10}}$. As indicated above, the other three members of RP-structured ruthenates, $\hbox {Sr}_2\hbox {RuO}_4$, $\hbox {Sr}_3\hbox {Ru}_2\hbox {O}_7$ and $\hbox {SrRuO}_3$, also show kinks within the same energy scales which were proposed to originate from coupling between electrons and phonons in these compounds^55,59^. In terms of crystal structure, these compounds belong to the layered perovskite oxide family, which are constituted of oxygen octahedra connected by oxygen atoms. The vibration of oxygen atoms, together with various tilts and distortions of similar oxygen octahedra leads to considerable phonon modes. When such phonon modes couple with electrons, renormalization effects (Supplementary Figure  S4a,b) with cut-off binding energies occur together with changes in the scattering rate near-$E_F$; thus leading to the formation of kinks in electronic band dispersions. Therefore, we suggest that electron-phonon interactions may be ubiquitous in $\hbox {Sr}_{n+1}\hbox {Ru}_{{n}}\hbox {O}_{3n+1}$ compounds impacting the physical properties of these compounds.

We would like to point out, on the other hand, that since the compound $\hbox {Sr}_4\hbox {Ru}_3\hbox {O}_{{10}}$ is a structurally distorted ferromagnet with $\hbox {RuO}_6$ octahedra rotations^8^, sensitive spin-phonon coupling is also expected in this compound. Notably, the 380 $\hbox {cm}^{-1}$ $B_{1g}$ phonon mode, reported previously to have a structural contribution to magnetic order in $\hbox {Sr}_4\hbox {Ru}_3\hbox {O}_{{10}}$^62^, corresponds roughly to the ARPES kink of 45 meV. This suggests the presence of sensitive spin-lattice coupling and points to the possibility that some of the ARPES resolved kinks are a manifestation of coupling between structural and magnetic properties in this system.

## Flat bands close to the Fermi-level in band structure of $\hbox {Sr}_{{4}}\hbox {Ru}_{{3}}\hbox {O}_{{10}}$

**Figure 4:**
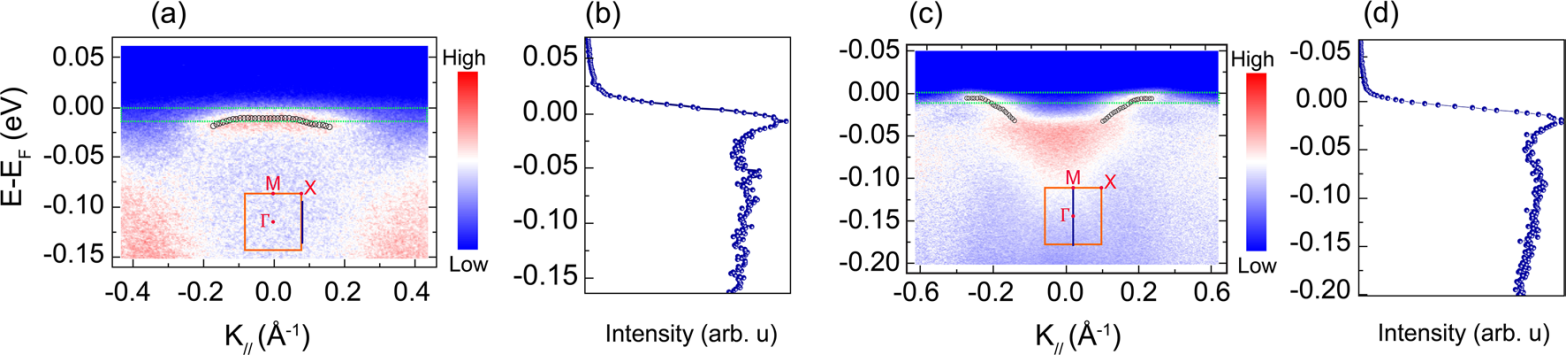
ARPES two dimensional cuts obtained in the proximity of $E_F$ measured using a photon energy of (**a**) 37 eV and (**c**) 60 eV for the sample $A_2$. The blue vertical line in the first BZ in the insert shows the direction in which the cuts were acquired. The black open symbol plotted on top of the flat bands of interest, (**a**) $\alpha _2$ and (**c**) $\delta$, correspond to the binding energy versus momentum quasiparticle peaks obtained by fitting several EDCs taken from the $\gamma$ and $\delta$ bands with a Gaussian line shape. (**b**,**d**) Sharp peaks in the DOS located in a small energy window ($\lesssim 8$ meV) around the Fermi-level, as indicated by light green dashed rectangles.

Finally, we reserve this last section to discuss the possible microscopic origin of the metamagnetic transition due to fine structure, within an energy range relevant for the Zeeman effect, present in the near-$E_F$ electronic structure of $\hbox {Sr}_4\hbox {Ru}_3\hbox {O}_{{10}}$. It is an itinerant type of metamagnetism that is relevant in this system, which has also been reported in 3*d* and $f-$electron systems^64,65^. Itinerant metamagnetism is often predicted for electron systems with a two-dimensional DOS that has a logarithmically divergent vHS^66–68^. The presence of such a vHS in the DOS renders the material magnetically unstable such that the application of a magnetic field induces a metamagnetic transition by shifting the vHS across $E_F$. Previous band structure calculations and ARPES experiments have proposed this itinerant metamagnetism model to explain the complex band structure of the parent compound $\hbox {Sr}_3\hbox {Ru}_2\hbox {O}_{{7}}$^32–34^. According to these works, it is the presence of such sharp peaks in the near-$E_F$ DOS (i.e., within a few meV from $E_F$) that are the electronic origin of magnetic fluctuations present in $\hbox {Sr}_3\hbox {Ru}_2\hbox {O}_{{7}}$^34,35^. The natural cause of sharp peaks in the DOS is the presence of flat bands close to $E_F$. The same scenario could also be valid for $\hbox {Sr}_4\hbox {Ru}_3\hbox {O}_{{10}}$ given the close similarity between these two systems. However, we point out that our ARPES data on near-$E_F$ flat bands within an energy range relevant for the Zeeman effect are preliminary, therefore the discussion presented below remains at a speculative level.

We have identified in different ARPES spectra of $\hbox {Sr}_4\hbox {Ru}_3\hbox {O}_{{10}}$ two flat bands of Ru 4*d* that present a complex DOS within a few meV of $E_F$, which is the energy scale relevant for metamagnetism (Fig. 4a,c). The $\alpha _2$ and $\delta$ bands were observed to have a large DOS just close to $E_F$, a situation that is susceptible to give rise to vHS in the DOS (Fig. 4b,d). The dispersion of the quasiparticle peak of these bands are very narrow and confined within a narrow energy window ($\lesssim 8$ meV) around $E_F$. This motivates a speculative discussion on the electronic origin of the metamagnetism in $\hbox {Sr}_4\hbox {Ru}_3\hbox {O}_{{10}}$. It is possible that through the application of an external magnetic field, the $\alpha _2$ and $\delta$ FS sheets may be spin polarized at a field of $T_M\approx 2.5$ T, where $T_M$ is the magnetic field at which the metamagnetic transition in $\hbox {Sr}_4\hbox {Ru}_3\hbox {O}_{{10}}$ is observed along the *ab*-plane. The $\alpha _2$ and $\delta$ FS sheets could then jump discontinuously over $E_F$ and give rise to metamagnetic behaviour observed in this compound.

Nonetheless, it is important to emphasize the effect of resolution in order to resolve such vHS in the near-$E_F$ DOS. To resolve narrow bands in the band structure of $\hbox {Sr}_3\hbox {Ru}_2\hbox {O}_{{7}}$, Tamai et al. used an ARPES experimental energy resolution of 5.5 meV. This was of great importance in their work as some carrier sheets in their data showed an occupied band width of only 5 meV^34^. An energy resolution greater than 10 meV or the presence of an impurity scattering contribution of this order to the linewidth of the resolved peaks would have rendered it impossible to resolve the narrow flat bands at $E_F$ and consequently not detect the vHS in the near-$E_F$ DOS. In our experiment on $\hbox {Sr}_4\hbox {Ru}_3\hbox {O}_{{10}}$, the FWHM of the sharpest peak, extracted through EDC fitting, was $\sim 21$ meV. Thus, further ARPES experiments with a better energy resolution and band structure calculations are necessary to ascertain the role of these two flat bands, $\alpha _2$ and $\delta$, as potential candidates that could be responsible for the appearance of metamagnetism in $\hbox {Sr}_4\hbox {Ru}_3\hbox {O}_{{10}}$, as has already demonstrated in its parent compound^34,35^.

## Conclusion

In summary, we have systematically investigated the electronic band structure of $\hbox {Sr}_4\hbox {Ru}_3\hbox {O}_{{10}}$ using synchrotron based-ARPES. This study has provided the first information on the near-$E_F$ band dispersions and FS of $\hbox {Sr}_{{4}}\hbox {Ru}_3\hbox {O}_{{10}}$ and the effect of changing different matrix elements on electronic band dispersions, as well as electronic correlation effects present in this compound.

A detailed analysis of different ARPES cuts showed that four bands are crossing the Fermi-level. These bands give rise to four FS sheets and the careful analysis of each FS sheet showed that $\delta$ and $\gamma$ are hole-like while the $\alpha _1$ and $\alpha _2$ are electron-like FS sheets. Exploiting different matrix elements, by using different photon energies and changing light polarization, it was possible to better discern some features in band dispersions and FS maps of $\hbox {Sr}_4\hbox {Ru}_3\hbox {O}_{{10}}$. For example, using different light polarizations (LHP and LHP), it was possible to resolve the separation of $\alpha _1$ and $\alpha _2$ dispersing states in LVP, while in LHP these bands seemed to form one single band. Five different kink features of energies ranging from 30 to 69 meV were resolved. These kink structures were found to be approximately equal to the energies of the vibrational states detected by Raman spectroscopy and lattice dynamic calculations studies. This observation, together with comparison to kink structures reported in other three members of RP-structured ruthenates, demonstrate that the kinks in $\hbox {Sr}_4\hbox {Ru}_3\hbox {O}_{{10}}$ originate from the strong electron–phonon coupling. We have also discussed the possible ubiquity of electron–phonon coupling in $\hbox {Sr}_{n+1}\hbox {Ru}_{{n}}\hbox {O}_{3n+1}$ systems. Finally, these ARPES data show two flat bands, $\alpha _2$ and $\delta$, that exhibit a complex DOS closer to the Fermi-level, suggesting that these two bands are the most likely bands that will give rise to vHS in the near Fermi-level DOS, a situation which is favorable for magnetic instabilities in $\hbox {Sr}_4\hbox {Ru}_3\hbox {O}_{{10}}$ material. We like to finish by also pointing out that this work is inline with current efforts to have in the near future the first African synchrotron light-source built on the African continent^69–71^, which is expected to boost research output for African scientists and their international colleagues conducting experimental research related to or enabled by synchrotron light-sources.

## Supplementary information

Supplementary Information.

## Data Availability

The data that support the findings of this study are available from P. N, E. C and B. P. D. upon reasonable request.
