# Supplementary material for: Fermi surface and kink structures in Sr4Ru3O10 revealed by synchrotron-based ARPES
Source: Sci Rep. 2020 Dec 3;10:21062. doi: 10.1038/s41598-020-77845-x (PMC7712785; doi:10.1038/s41598-020-77845-x)
Supplement: Supplementary file 1 — Supplementary Information. [file 41598_2020_77845_MOESM1_ESM.pdf]

# Supporting Information:

## Fermi surface and kink structures in $Sr_4Ru_3O_{10}$ revealed by synchrotron-based ARPES

Prosper Ngabonziza[1,2], Emanuela Carleschi[2], Volodymyr Zabolotnyy[3], Amina Taleb-Ibrahimi[4], François Bertran[4], Rosalba Fittipaldi[5,6], Veronica Granata[5,6], Mario Cuoco[5,6], Antonio Vecchione[5,6], Bryan Patrick Doyle[2]

[1]Max Planck Institute for Solid State Research, D-70569 Stuttgart, Germany

[2]Department of Physics, University of Johannesburg, P.O. Box 524 Auckland Park 2006, Johannesburg, South Africa

[3]Physikalisches Institut, Julius-Maximilians-Universität Würzburg, Am Hubland,97074 Würzburg,Germany

[4]Synchrotron SOLEIL, L'Orme des Merisiers, Saint-Aubin-BP48, 91192 Gif-sur-Yvette, France

[5]CNR-SPIN Salerno, Via Giovanni Paolo II, 84084 Fisciano, Italy

[6]Department of Physics, University of Salerno, Via Giovanni Paolo II, 84084 Fisciano, Italy

# Determination of the Fermi Level and Experimental Energy Resolution

To determine the Fermi-level ($E_F$) for each sample measured and for each incoming photon energy used, several energy dispersive curves (EDCs) taken at different specific momenta were fitted with a Fermi-Dirac distribution function:

$$f(E) = \frac{1}{1 + \exp\left(\frac{E_{kin} - E_F}{K_B T}\right)};$$

added to a linear background. Here, $E_{kin}$ is the kinetic energy, $K_B$ the Boltzmann constant and $T$ the temperature. The linear background was introduced to take into account of the electrons produced by second order light coming from the undulator of the synchrotron and transmitted by the beamline optics. Figure S1a shows equally spaced energy distribution curves taken at different momenta for an ARPES band dispersion acquired at a photon energy of 60 eV. To obtain the Fermi level $E_F$, a single EDC which has a Fermi-Dirac distribution like line-shape (the red curve) was taken from these equally spaced EDCs. Fitting this specific EDC [Figure S1b], the Fermi-level value of $E_F = 55.903$ eV is extracted. To check the accuracy of the obtained value of $E_F$, in each case 10 different EDCs have been fitted, and the change in the value of $E_F$ was found to be approximately ± 0.5 meV. The obtained $E_F$ was used to set the binding energy scale in band dispersions and in energy distribution curves, using the expression $E_B = E_F - E_{kin}$.

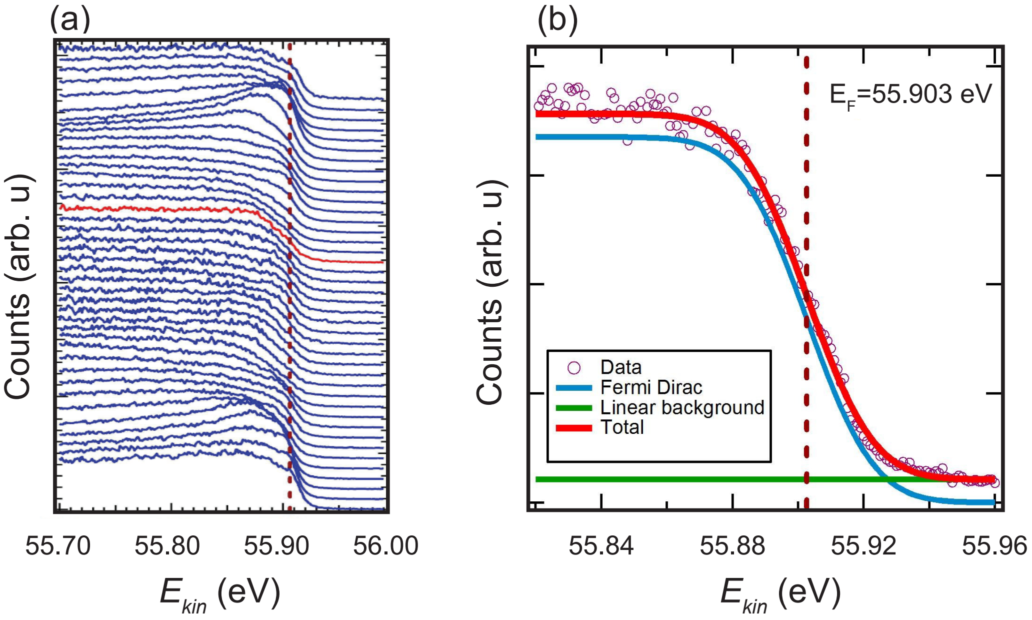


**FIG. S1**. (a) Representative series of equally spaced energy dispersion curves (EDCs) extracted from an ARPES cut at different specific momenta for the sample $A_2$. The EDC spectrum in red is reported separately in purple open symbols (b) together with the overall fitting with a Fermi-Dirac distribution function added to a linear background to extract the Fermi energy. The blue curve is the Fermi-Dirac distribution function, the green line is the linear background while the red curve is their addition which is the fit to the data.

## Analysis of the Fermi Surface Sheets

Analysis of several band dispersions, taken at different locations in the first BZ, allowed us to determine the electron or hole character of the four FS sheets. Figure S2a-c show ARPES cuts measured with a photon energy of $60$ eV for the sample $A_1$. The black open circles on top of the bands of interest, $\gamma, \alpha$ and $\delta$ are MDC derived dispersions of these bands obtained by fitting several MDCs with Lorentzian functions. From these derived dispersions, it is clear that the $\gamma$ and $\delta$ bands have an inverted parabola-like dispersions as illustrated by the green parabola at the top of these bands; whereas the $\alpha$ bands have upward parabola-like behaviours. Thus, the $\gamma$ and $\delta$ bands will give rise to hole FS sheets whereas the the $\alpha$ bands will give rise to electron FS sheets. Table S1 summarizes the extracted effective masses in the free electron approximation and the character (hole or electron).

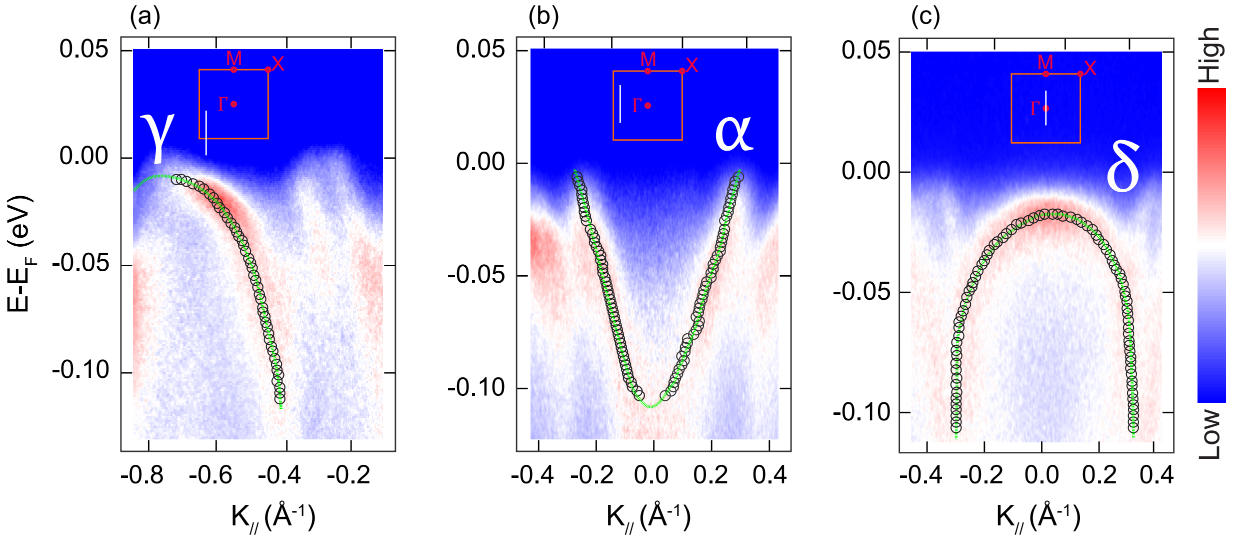


**FIG. S2**. Investigation of electron and hole character of the ARPES resolved FS sheets of $Sr_4Ru_3O_{10}$ for the (a) $\gamma$ band, (b) $\alpha$ band, taken under conditions where the $\alpha_1$ and $\alpha_2$ seem like one band, and (c) $\delta$ band. The black open circles at the top of bands are MDCs derived dispersion of these bands. The dispersion of these bands show parabola-like behaviour (green dashed lines on top of the bands of interest). (Inset) The white vertical lines indicate the position and direction in which the cuts have been acquired with respect to the first BZ (square orange rectangle). The spectrum in (a) is from the sample $A_2$, whereas the spectra in (b) and (c) are from the sample $A_1$.

**TABLE S1**. Characteristics of the FS sheets of $Sr_4Ru_3O_{10}$. The character of each pocket is indicated in square brackets, $h^+$ for hole pockets and $e^-$ for electron pockets. The effective masses of each FS sheet are also given. The effective mass of the $\delta$ FS sheet was not determined because the $\delta$ band is so broad. It was difficult to find its position at the Fermi-level and its $v_f$, but it gave a spectral weight at $E_F$.

| Character FS sheets | $\delta(h^+)$ | $\alpha_1(e^-)$ | $\alpha_2(e^-)$ | $\gamma(h^+)$ |
|---|---|---|---|---|
| Effective mass | – | 0.81 | 0.41 | 2.95 |

# Photoemission Matrix Elements

The expression of the measured photoemission intensity can be written as:

$$I(k,\omega) = I_0(k,\omega,A)f(\omega)A(k,\omega);$$

where $f(\omega)$ and $A(k,\omega)$ are the Fermi-Dirac distribution function and the spectral function, respectively. To discuss the effect of the matrix elements on photoemission spectra, one looks at the function $I_0(k,\omega,A)$ which is proportional to the square of the matrix elements $|M^{\mathbf{k}}_{f,i}|^2$. It modulates intrinsic intensities according to the geometric experimental constraints, and it depends on photon energy,

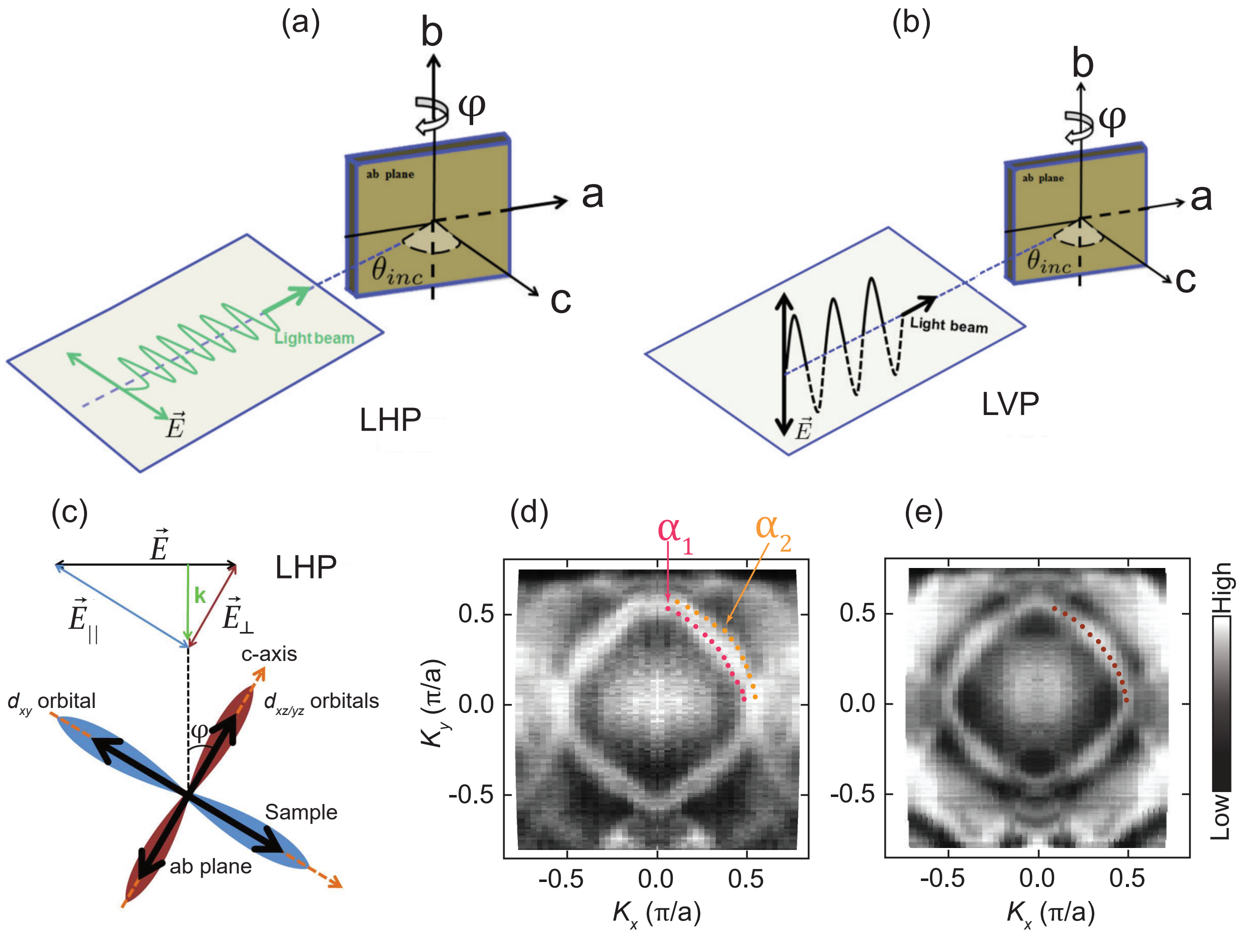


**FIG. S3**. Matrix element effect using different light polarizations. Illustrative sketch showing the difference between light coming in (a) linear horizontal polarization (LHP) and (b) linear vertical polarization (LVP) with respect to the experimental geometry. (c) Schematic illustration of LHP light impinging on a sample with $d_{xy}$ and $d_{xz/yz}$ oriented orbitals. The parallel component of the electric field excites both in-plane $d_{xy}$ and out-of plane orbitals $d_{xz/yz}$, depending on the value of the polar angle $\varphi$. However, the perpendicular component of the electric field excites only in-plane orbitals $d_{xy}$. Fermi surface maps measured with a photon energy of 60 eV for the sample $A_1$ in (d) LVP and (e) in LHP. The markers plotted on top of the FS sheets are FS contours extracted from fitted MDC curves for the $\alpha_1$ and $\alpha_2$ bands. In LHP, the two bands cannot be distinguished.

electron momentum and light polarization [1].

During the ARPES experiment at the CASSIOPÉE beamline, spectra were acquired at different photon energies using two polarizations: linear horizontal polarization (LHP) and linear vertical polarization (LVP). This was done to take advantage of different matrix elements and also to determine the symmetry and character of the near-$E_F$ dispersing states. Figure S3a-b show the polarization configurations exploited in the ARPES experiment with respect to the experimental geometry. The manipulator used at CASSIOPÉE only allows rotation of the sample around the vertical axis of the manipulator angle $\varphi$. This is why pre-orientation of the sample on the sample holder was necessary. Changing the

sample orientation by varying $\varphi$ within a specific light polarization, it possible to probe in-plane and out-of-plane bands. When the light is in LHP [Figure S3a], the electric field $\vec{E}$ is oscillating in the same plane as the orbit of the electrons in the ring and lies in the plane determined by the $a$ and the $c$ axes of the sample; while for light in LVP [Figure S3b], the electric field is oscillating in the perpendicular plane to the orbit of the electrons and it is parallel to the $a$ axis. This implies that when light is in LVP, the electric field $\vec{E}$ is always in the $ab$ plane; while in LHP, the electric field $\vec{E}$ has two components: an out-of-plane component, given by $\vec{E}_{\perp}$, and an in-plane component, given by $\vec{E}_{\parallel}$. Thus, by rotating the sample with an angle $\varphi$ around the vertical axis, it is possible to probe both in-plane bands (for $\varphi \simeq 0$) and out-of-plane bands (for higher values of $\varphi$) when incoming light is in LHP. However, with LVP, it is only possible to probe in-plane bands. This is evidenced in a more clear way by the sketch shown in Figure S3c. For simplicity, we consider a general sample with $d_{xy}$ and $d_{xz/yz}$ orbitals that is illuminated by LHP light. In this configuration with light in LHP, it is possible to probe both in-plane band character $(d_{xy}, d_{x^2-y^2})$ and out-of-plane band character $(d_{xz}, d_{yz})$ by changing the polar angle $\varphi$. However, for in-coming light in LVP, it is only possible to probe in-plane bands $(d_{xy}, d_{x^2-y^2})$ as rotating the sample by an angle $\varphi$ around the vertical axis will yield no information on the near-$E_F$ out-of-plane dispersing states. Polarization dependence of incoming light was exploited in the ARPES experiment to probe symmetry properties of the electronic states of $Sr_4Ru_3O_{10}$. Figure S3d-e show two FS maps taken using different polarizations of incoming light. For the FS map in LVP [Figure S3d], two FS sheets ($\alpha_1$ and $\alpha_2$) are well resolved and they are clearly separate. In LHP, these two FS sheets are joined and form one FS sheet on the FS map [Figure S3e].

# Correlated Effect in the Band Structure of $Sr_4Ru_3O_{10}$

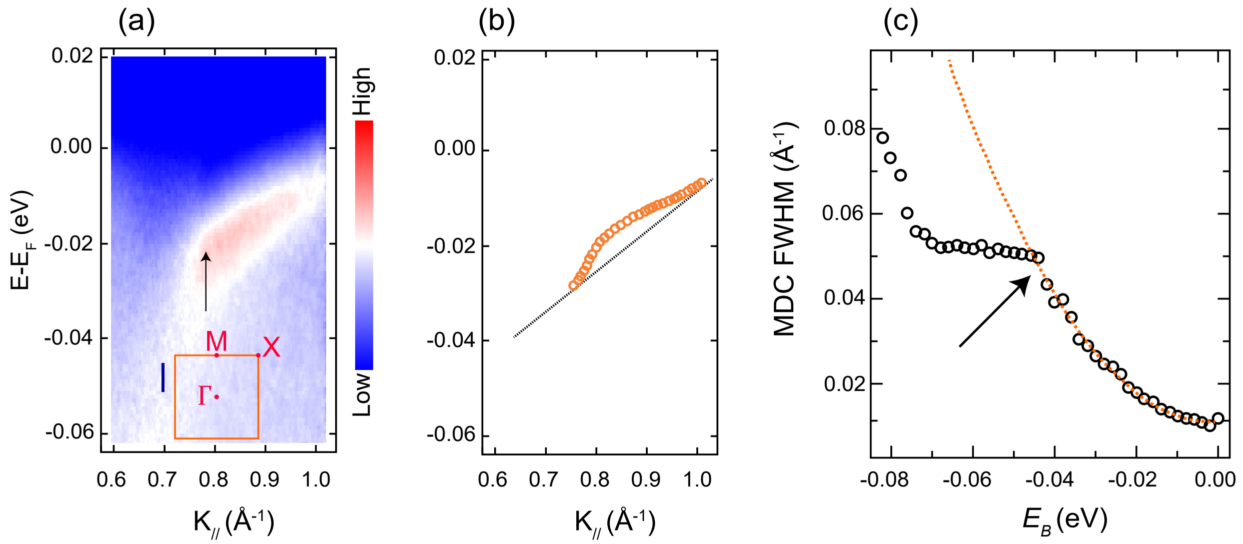


**FIG. S4**. Renormalised bands in the electronic band dispersions of $Sr_4Ru_3O_{10}$ measured with a photon energy of 60 eV in LHP, in the direction parallel to the $\Gamma - M$ line **for the sample** $A_2$. The blue vertical lines indicate the position and direction in which the cuts have been acquired with respect to the first BZ (square orange rectangle in inset). The black arrows point to the band of interest. (b) The orange open circles are peak maxima obtained by fitting MDCs extracted from the corresponding ARPES cut in (a). The black straight dashed-line represent band dispersions of a non-interacting system (the bare band dispersion). (c) A representative momentum (MDC) full width half maximum (FWHM) for the band of interest (the kink in Figure 3b in the Main Text) plotted versus the binding energy of the peak. The dashed red curve represents a quadratic fit to the low-energy data. The arrow marks the position of the kink.

**TABLE S2**. ARPES kink energies from this study and previously reported phonon modes from Raman spectroscopy and lattice dynamic calculations (LDCs) [2]. The symmetry of the Raman modes, either $B_{1g}$ or $A_g$, is indicated. The comparison shows that the kink energies resolved from ARPES data are approximately equal to the energy of the phononic modes revealing a compatibility between their energy scales.

| ARPES (meV) | $30 \pm 3$ | $40 \pm 2$ | $45 \pm 2$ | $65 \pm 1$ | $69 \pm 4$ |
|---|---|---|---|---|---|
| Raman Spectroscopy (meV) | 29.15 $A_g$ | 38.08 $B_{1g}$ | 47.1 $B_{1g}$ | – – | 72.95 $A_g$ |
| LDCs (meV) | 28.66 $A_g$ | 40.5 $B_{1g}$ | 43.92 $B_{1g}$ | 63.52 $A_g$ | 68.48 $A_g$ |

# Remarks on Early Specific Heat, Susceptibility and Resistivity Measurements for $Sr_4Ru_3O_{10}$

In the work by X.N. Lin *et al.*, the zero field specific heat experiment showed a weak anomaly at the Curie temperature while no anomaly was observed at the metamagnetic transition temperature [3]. The magnetic field dependence of the specific heat revealed a large variety of anomalies with growing specific heat by increasing the field and abrupt jumps at critical fields applied in the $ab$-plane and along the $c$-axis [4]. In the work by Cao *et al.*, they also found a strong anisotropy in the magnetotransport with non-standard temperature dependence highlighting a possible non-trivial role of critical magnetic fluctuations and electron correlations [4]. The first susceptibility data were reported by M.K. Crawford *et al.*, confirming the ferromagnetic/metamagnetic nature of the magnetism in the $Sr_4Ru_3O_{10}$ system [5]. The magnetic structure has been further refined by neutron scattering experiments with the aim to set out the structural and magnetic interrelation as well as to establish the magnetic and orbital pattern within the unit cell [6–8]. In addition, several authors have also studied the resistivity characteristics of $Sr_4Ru_3O_{10}$ single crystals. For example, Mao *et al.* [9] and Fobes *et al.* [10] have investigated the resistivity as function of magnetic field and temperature. They observed unusually transport behaviours around the metamagnetic transition. Their results showed sharp steps in the in-plane resistivity $\rho_{ab}$ for downward field sweeps between 1.75 T and 2.5 T and a remarkable non-metallic temperature dependence within this transition regime.

# Possible Low Energy Kink

In laser-based ARPES data on the parent ruthenate compound $Sr_2RuO_4$, Akebi and co-workers report several kinks in the low energy band dispersion of this material [11]. One such kink at very low energy ($\sim$ 8 meV) is attributed to magnetic excitations in relation to a peak found by inelastic polarised neutron scattering at 10 to 15 meV in the DOS of the material [12]. It is not immediately clear that one can transfer such observations to $Sr_4Ru_3O_{10}$ since one deals with a substantially different electronic system having triple Ru-O layers in the unit cell and a ferromagnetic ground state, thus already in a broken symmetry phase if compared to the paramagnetic state of $Sr_2RuO_4$. Moreover, there are no available inelastic neutron scattering data for $Sr_4Ru_3O_{10}$ which can guide the identification of the low-energy electron-mode coupling on the surface in the context of magnetic excitations. On the other hand, one can argue that in the ferromagnetic state with a critical temperature of the order of 100 K, the characteristic excitation energy of the spin-waves could be about 8 to 10 meV. With

respect to having inelastic modes due to incommensurate or ferromagnetic fluctuations as in $Sr_2RuO_4$, it is therefore plausible that the lowest energy kink arises from the coupling between the electron-mode and the spin-wave excitations. This issue might be important to assess the nature of the magnetic ground state on the surface of the $Sr_4Ru_3O_{10}$ especially in view of the lack of observed spin-split bands. Considering a possible connection with the flat band in Figure 4 in the main text, while the energy scales are comparable, it is difficult to argue about the occurrence of an electron-magnon coupling so strong that is able to induce spin polarons that are substantially non-dispersive. It is more plausible to expect that the Coulomb interaction is relevant to drive a significant renormalization of the bandwidth and in addition to that the coupling with magnons can make a weak kink in the dispersion. For the sake of completeness, due to the intricate magneto-structural effects, one cannot exclude that magnons and phonons cooperate at that energy scale to increase the effective mass of the electron modes.

## References


[1] A. Damascelli, *Probing the electronic structure of complex systems by ARPES*, Phys. Scr. **T109**, 61 (2004).

[2] M. N. Iliev, V. N. Popov, A. P. Litvinchuk, M. V. Abrashev, J. Bäckström and Y. Y. Sun, R. L. Meng, C. W. Chu, *Comparative Raman studies of $Sr_2RuO_4$, $Sr_3Ru_2O_7$ and $Sr_4Ru_3O_{10}$*, Physica B: Condens. Matter **358**, 138 (2005).

[3] X. N. Lin, V. A. Bondarenko, G. Cao, J. W. Brill, *Specific heat of $Sr_4Ru_3O_{10}$*, Solid State Commun., **130**, 151 (2004).

[4] G. Cao, S. Chikara, and J. W. Brill, P. Schlottmann, *Anomalous itinerant magnetism in single-crystal $Sr_4Ru_3O_{10}$: A thermodynamic and transport investigation* , Phys. Rev. B, **75**, 024429 (2007).

[5] M. K. Crawford, R. L. Harlow, W. Marshall, Z. Li, G. Cao, R. L. Lindstrom, Q. Huang, J. W. Lynn, *Structure and magnetism of single crystal $Sr_4Ru_3O_{10}$: A ferromagnetic triple-layer ruthenate,* Phys. Rev. B **65**, 214412 (2002).

[6] F. Forte, L. Capogna, V. Granata, R. Fittipaldi, A. Vecchione, M. Cuoco, *Suppression of the orbital magnetic moment driven by electronic correlations in $Sr_4Ru_3O_{10}$*, Phys. Rev. B **100**, 104440 (2019).

[7] V. Granata, L. Capogna, F. Forte, M.-B. Lepetit, R. Fittipaldi, A. Stunault, M. Cuoco, A. Vecchione, *Spin-orbital nature of the high-field magnetic state in the $Sr_4Ru_3O_{10}$*, Phys. Rev. B **93**, 115128 (2016).

[8] V. Granata, L. Capogna, M. Reehuis, R. Fittipaldi, B. Ouladdiaf, S. Pace, M. Cuoco, A. Vecchione, *Neutron diffraction study of triple-layered $Sr_4Ru_3O_{10}$*, J. Phys.: Condens. Matter **25**, 056004 (2013).

[9] Z. Q. Mao, M. Zhou, J. Hooper, V. Golub, C. J. O'Connor, *Phase separation in the itinerant metamagnetic transition of $Sr_4Ru_3O_{10}$*, Phys. Rev. Lett. **96**, 077205 (2006).

[10] D. Fobes, M. H. Yu, M. Zhou, J. Hooper, C. J. O'Connor, M. Rosario, and Z. Q. Mao, *Phase diagram of the electronic states of trilayered ruthenate* $Sr_4Ru_3O_{10}$, Phys. Rev. B **75**, 094429 (2007).

[11] S. Akebi, T. Kondo, M. Nakayama, K. Kuroda, S. Kunisada, H. Taniguchi, Y. Maeno, S. Shin, *Low-energy electron-mode couplings in the surface bands of* $Sr_2RuO_4$ *revealed by laser-based angle-resolved photoemission spectroscopy,* Phys. Rev. B **99**, 081108(R) (2019).

[12] P. Steffens, Y. Sidis, J. Kulda, Z. Q. Mao, Y. Maeno, I. I. Mazin, M. Braden, *Spin Fluctuations in* $Sr_2RuO_4$ *from Polarized Neutron Scattering: Implications for Superconductivity,* Phys. Rev. Lett. **122**, 047004 (2019).
